# Consultation report – considerations for a regulatory pathway for bivalent *Salmonella* Typhi/Paratyphi A vaccines for use in endemic countries

**DOI:** 10.1016/j.vaccine.2025.127189

**Published:** 2025-05-22

**Authors:** Ana Belen Ibarz-Pavon, Marie-Christine Bielsky, Rubina Bose, Marco Cavaleri, John A. Crump, Joachim Hombach, David C. Kaslow, Farhana Khaman, Calman A. MacLennan, Kirsty Mehring-LeDoare, Andrew J. Pollard, Firdausi Quadri, Jacob John, Annelies Wilder-Smith

**Affiliations:** a World Health Organization, Department of Immunization, Vaccines and Biologicals, Geneva, Switzerland; b Medicines and Healthcare products Regulatory Agency (MHRA), London, UK; c Central Drugs Standard Control Organization (CDSCO), India; d European Medicines Agency, Netherlands; e Centre for International Health, University of Otago, Dunedin, New Zealand; f Food and Drug Administration, Center for Biologics Evaluation and Research, Silver Spring, MD, USA; g International Centre for Diarrhoeal Disease Research, Dhaka, Bangladesh; h Bill & Melinda Gates Foundation, Enteric & Diarrheal Diseases, 440 5th Ave N, Seattle, WA 98109, USA; i Jenner Institute, Nuffield Department of Medicine, University of Oxford, Oxford, UK.; j City St. George's, University of London, London, UK; k Oxford Vaccine Group, Department of Paediatrics, University of Oxford, and The NIHR Oxford Biomedical Research Centre, Oxford, UK; l Christian Medical College, Vellore, India

**Keywords:** Enteric fever, *Salmonella* Paratyphi A, Paratyphoid fever, Vaccines, Phase 3 study, Regulatory pathway

## Abstract

Enteric fever caused by *Salmonella enterica* serovars Typhi and Paratyphi A and, to a lesser extent, *S.* Paratyphi B and C, remains a significant cause of mortality and morbidity in resource-constrained settings. Typhoid conjugate vaccines (TCVs) protect against *S.* Typhi but no vaccine to date protects against paratyphoid fever. There are several bivalent *S.* Typhi/Paratyphi A products in development; however, the low incidence of paratyphoid fever in many settings limits the feasibility of phase 3 efficacy studies. Two bivalent vaccines adding the *S.* Paratyphi A-specific O:2 lipopolysaccharide conjugated to a protein carrier to TCV constructs have successfully completed phase 1 studies and will progress rapidly in their development.

The WHO's Product Development for Vaccines Advisory Committee (PDVAC) endorsed a regulatory pathway for a bivalent *S.* Typhi**/**Paratyphi A vaccine that contemplates demonstrating protective efficacy against *S.* Paratyphi A infection in a controlled human infection model (CHIM). Since the use of CHIM data *in lieu* of phase 3 efficacy studies and to identify markers of immune protection is not yet widely accepted by regulatory bodies, the WHO organized a consultation with vaccine developers, manufacturers, and regulators. The purpose of the meeting was to discuss the feasibility and considerations for the licensure of a bivalent *S.* Typhi/Paratyphi A vaccine. The aim of the consultation was to gain alignment among key stakeholders and facilitate the pathway to licensure in endemic countries.

## Highlights

• Paratyphoid fever due to *Salmonella Paratyphi* A is the second most prevalent cause of enteric fever, particularly in South East Asia.
• Pathway to licensure for bivalent vaccines is unlikely to follow a classic Phase 3 efficacy trial as these are not currently feasible.
• In July 2024, WHO convened a regulatory science meeting to discuss the acceptability of an alternate regulatory pathway.
• Academic researchers, vaccine developers and manufacturers, and regulatory authorities discussed a regulatory pathway that uses CHIM to establish vaccine efficacy.
• We provided recommendations to address the specific considerations of such regulatory pathway.

## 1 Introduction

Enteric fever, caused by typhoidal strains of *Salmonella enterica* (Typhi and Paratyphi A, B, and C), is a community-acquired infection primarily spread through contaminated food and water. [[1], [2], [3]]. Risk factors for enteric fever include lack of access to safe water, unsafe food, unimproved sanitation facilities, residence in unsanitary, over-crowded conditions, and young age, notwithstanding the substantial disease burden among older age groups [[4], [5], [6]]. The disease has an incubation period of 7–14 days, and often presents as fever sometimes accompanied by general malaise, vomiting, and abdominal pain, making it indistinguishable from other febrile illnesses [7]. Diagnosis requires microbiological testing using blood culture in a well-resourced laboratory, which is not always available in low- and middle-income countries (LMICs) where the disease is endemic. Widespread use of poorly sensitive and specific tests such as the Widal test also add to the problems of rapid detection and diagnosis [8]. Paratyphoid fever is clinically indistinguishable from typhoid fever, and both are frequently misdiagnosed as other febrile illnesses. Consequently, enteric fever may be misclassified, and the role of paratyphoid fever as a cause of enteric fever overlooked. If untreated, serious complications such as peritonitis, gastrointestinal haemorrhage, and intestinal perforation can occur, leading to death [9,10]. In 2021 there were globally an estimated >9.3 million illnesses and > 107,000 deaths from enteric fever, and the disease resulted in ∼1.8 million disability-adjusted life years (DALYs). In contrast to typhoid fever, which is endemic in Asia and sub-Saharan Africa, paratyphoid fever is mostly confined to south and south east Asia, yet, *S.* Paratyphi A was responsible for 2,166,062 (23.2 %) of enteric fever illnesses, 14,126 (13,1 %) deaths, and 1,011,841 (12.5 %) DALYs in 2021 [11].

Timely administration of effective antimicrobials is central to prevention severe outcomes from enteric fever. Traditionally, chloramphenicol, amoxicillin, and trimethoprim-sulfamethoxazole have been the recommended as first-line treatment. However, misuse of antimicrobials has led to the emergence of antimicrobial resistant (AMR) typhoidal *Salmonellae* that no longer respond to these drugs. Multidrug resistance (MDR) in typhoidal *Salmonellae*, defined as non-susceptibility to all three first-line antimicrobials, is prevalent in Asia and sub-Saharan Africa, while MDR *S.* Paratyphi A remains less common with an estimated global prevalence of 0.2 % in 2019 [12]. The use of fluoroquinolones as an alternative treatment has led to a rapid spread of fluoroquinolone non-susceptibility (FQNS) in typhoidal *Salmonella* serovars, and more than 95 % of *S.* Paratyphi A isolates are FQNS [12,13]. In 2016, an extensively-drug resistant (XDR) *S.* Typhi, an MDR strain additionally resistant to third-generation cephalosporins and ciprofloxacin, emerged in 2016 in Sindh, Pakistan [14,15], and has since become a well-established cause of typhoid fever throughout the country [16,17]. The strain was subsequently identified internationally, mainly imported through returning travellers from Pakistan, and remains a public health threat [17,18]. While *S.* Paratyphi A XDR strains have not been identified to date, timely and robust laboratory-based surveillance of paratyphoid fever, and the development of an effective vaccine that protects against *S.* Paratyphi A in endemic settings are crucial to curtail the emergence and spread of AMR strains [12,[19], [20], [21], [22], [23], [24], [25]]. Vaccination has been shown to reduce antimicrobial prescriptions and help curb antimicrobial resistance (AMR) [[26], [27], [28], [29]]. The typhoid conjugate vaccine (TCV), which prevents enteric fever caused by *Salmonella* serovar Typhi, but offers no protection against *S.* Paratyphi A, was recommended for use by the WHO Strategic Advisory Group of Experts on Immunization (SAGE) in settings where enteric fever is endemic, and emphasized the need for its implementation in settings with high AMR prevalence [30,31]. Typbar-TCV, a conjugate construct of Vi-polysaccharide conjugated to tetanus-toxoid protein, was licensed in India in 2013. Protective efficacy for this vaccine was inferred from a similar construct of Vi-polysaccharide linked to the recombinant exoprotein A of *Pseudomonas aeruginosa* (Vi-rEPA), which was tested in Vietnam. The Vi-rEPA vaccine demonstrated a vaccine efficacy of 89 % among children aged 2–5 years four years after receiving two doses of the vaccine [[32], [33], [34]]. Licensure of Typbar-TCV was granted on the basis of favourable safety and immunogenicity when compared with a Vi-polysaccharide vaccine (Vi-PS) observed in a phase 3 trial conducted among ∼1000 individuals aged 2–45 years [35], and using the total anti-Vi IgG as an indicator of vaccine immunogenicity, as recommended by the WHO [36]. Licensure and WHO prequalification were supported by data obtained using a controlled human infection model (CHIM) study that showed a vaccine efficacy of 54.6 % (95 % CI 26.8–71.8), and demonstrated that the vaccine was able to induce a strong immune response [37]. Additionally, modelling studies predicted that the use of TCV could not only be a cost-effective measure resulting in significant disease reductions, but also reduce AMR typhoid fever cases by 16 %, preventing >53 million deaths in sub-Saharan Africa, and substantially reduce transmission and spread of MDR and FQNS [38,39].

In the absence of phase 3 efficacy data, SAGE relied on anti Vi-IgG as a surrogate marker of protection and modelling data for added value, as well as data from a CHIM to issue their recommendation on the use of TCVs. The CHIM evaluated the Typbar-TCV vaccine efficacy against a licensed Vi-Polysaccharide (Vi-PS) vaccine and used the meningococcal conjugate vaccine as a placebo. Typvar-TCV showed a vaccine efficacy of 54.6 % for TCV in comparison to a 52.0 % for the Vi-PS vaccine, and induced higher seroconversion rates than the Vi-PS comparator [37]. Subsequent TCVs were licensed and received WHO prequalification on the basis of immunobridging, by demonstrating a comparable immune response to Typbar-TCV [40]. While all four WHO-prequalified vaccines might differ on the carrier protein used, there is evidence that this does not impact the conjugate vaccine performance [41].

Field efficacy trials conducted in Malawi, Nepal, and Bangladesh validated the CHIM findings, demonstrating 79–85 % protection over two years [[42], [43], [44]]. However, waning immunity, particularly among children who were vaccinated before 2 years of age, has been reported in Bangladesh [45] and Pakistan [46], where the vaccine was introduced in response to an XDR *S.* Typhi outbreak in Hyderabad in 2018 [47]. This observation was also consistent in Malawi, where antibody waning was observed four years post-vaccination, and the lowest point estimates for vaccine effectiveness at this time point were observed in those vaccinated at a younger age [48].

There are currently no licensed vaccines to protect against paratyphoid fever. However, several bivalent products that would confer comprehensive protection against enteric fever caused by *S.* Typhi and *S.* Paratyphi A serovars are in development [49], with at least one product having completed and reported findings of their phase 1 study at the time of writing. Kulkarni and colleagues, from the Serum Institute India (SII), assessed the safety and immunogenicity of a bivalent vaccine that combines *S.* Typhi Vi capsular polysaccharide linked to tetanus toxoid, and the *S.* Paratyphi A O-specific lipopolysaccharide (O:2-LPS) conjugated to the diphtheria toxoid carrier protein. The study recruited 60 adults in India, and randomized participants to receive either Typbar-TCV or the bivalent product. The vaccine demonstrated that the immune responses against *S.* Typhi were comparable in both groups. In the intervention group, seroconversion for *S.* Paratyphi A anti-O:2-LPS serum IgG antibodies on day 29 and day 181 post-vaccination was observed in 100 % of participants, and serum bactericidal activity was observed in 93.3 % and 66.7 % of participants at day 29 and day 181 respectively. Additionally, the vaccine showed a good safety profile [50].

**Fig. 1:**
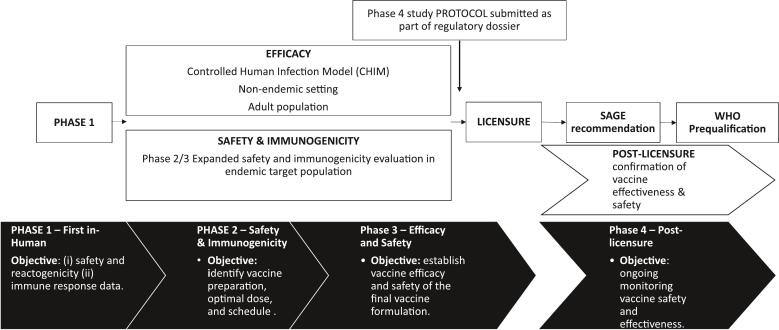
Clinical development pathway for conjugated bivalent *Salmonella Typhi*/Paratyphi A vaccines. Outlined in black at the bottom of the figure is the “classic” vaccine clinical development [81]. At the top, in white is the proposed clinical development pathway proposed for bivalent vaccines, where phase 2 & 3 studies to determine safety, immunogenicity, and noninferiority to the typhoid component take place concurrent to a CHIM study, leading to vaccine licensure and, eventually WHO prequalification.

Without phase 3 efficacy data or an established correlate of protection (CoP) for *S.* Paratyphi A infection to allow immunobridging, the pathway to licensure for bivalent vaccines remains a complex one [51]. Conducting phase 3 efficacy trials is challenging due to the low incidence of disease, the financing of large trials, and geographic restriction [51,52]. The WHO's Product Development for Vaccines Advisory Committee (PDVAC) supported an alternative licensure pathway involving CHIM studies in adults [53,54], phase 3 safety and immunogenicity trials in the target population in an endemic setting, and postmarketing effectiveness studies [55] (Fig. 1). Alignment with regulatory bodies, particularly, with those in endemic countries where a bivalent *S.* Typhi/Paratyphi A vaccine is likely to be used at a large scale, was identified as a priority by the WHO's technical advisory group on *Salmonella* vaccines (TAG-SV), and advisory group comprising academic researchers and vaccine developers with expertise on the disease, treatment, epidemiology and vaccinology of *Salmonella enterica* disease.

To facilitate acceptance of this development pathway, WHO convened a consultation with experts and regulators to discuss evidence and strategies for licensing a bivalent *S.* Typhi/Paratyphi A vaccine and to identify data gaps that could streamline the process. The meeting was held online in July 2024, and attended by WHO's TAG-SV, representatives from regulatory agencies from *S.* Paratyphi A endemic countries in Asia, and non-endemic countries in Europe, where CHIM studies are to be conducted, international regulatory agencies in Europe and the USA, and the African vaccine regulatory forum (AVAREF).

## 2 *Salmonella* Paratyphi A epidemiology, burden of disease, and antimicrobial resistance

According to the Institute of Health Metrics and Evaluation (IHME) Global Burden of Disease (GBD) 2021 study, there were 2.2 million illnesses globally and 14,1216 deaths globally attributed to paratyphoid fever in 2019, which accounted for 23.2 % and 13.1 % of enteric fever illnesses and deaths respectively. The GBD 2021 study also estimated that paratyphoid fever was responsible for >1 million disability adjusted life years (DALYs), accounting for 12.5 % of enteric fever DALYs. Over 95 % of illnesses occur in Asia, specifically, in South Asia, and the disease remains rare in Africa [11]. Prior to the introduction of TCV, several regional surveillance initiatives aimed at characterizing the burden of enteric with a focus on typhoid fever, were implemented. These initiatives include the Severe Typhoid in Africa (SETA) program, the Surveillance for Enteric Fever in Asia Project (SEAP), Surveillance for Enteric Fever in India (SEFI), and the Strategic Typhoid Alliance Across Africa and Asia (STRAATA) [[56], [57], [58]]. These programmes implemented a hybrid surveillance conducted in health facilities serving a well-defined and characterized population of approximately 100,000. Patients living in the catchment population area presenting to participating health facilities with patients with febrile illness were invited to enrol. Blood-culture confirmed typhoid fever illnesses were used to calculate crude and age-stratified incidence rates, and healthcare utilization surveys were used to adjust incidence rates by taking into consideration healthcare seeking behaviour. Further adjustments were made to account for probability of having a blood culture taken, and for blood culture sensitivity. As part of these initiatives, data on the incidence of paratyphoid fever in the study settings, as well as strain characteristics were generated. Since *S.* Paratyphi A is less common than *S.* Typhi, incidence estimates for *S.* Paratyphi A obtained from such studies are often imprecise, particularly, when stratified by narrow age groups [56].

The STRAATA study, which collected data from 2016 to 2018 [59], provided crude incidence estimates of *S.* Paratyphi A disease of 6 per 100,000 person-years (p-y) in Kathmandu, Nepal, and 42 per 100,000 p-y in Dhaka, Bangladesh. *Salmonella Paratyphi* A was not isolated in Blantyre, Malawi. Due to the low number of cases, it was not possible to accurately calculate adjusted incidence rates as was done for *S.* Typhi illnesses. However, the highest crude incidence of *S.* Paratyphi A across STRATAA sites in Asia was seen in children aged 5 through 9 years. The SEAP study [60] ran hybrid surveillance in Asia sites from 2016 to 2019. This study reported an overall adjusted incidence of paratyphoid fever of 128 *per* 100,000 p-y in Dhaka, Bangladesh. The two sites in Nepal yielded incidences of 46/100,000 and 81/100,000 p-y, and the incidences in the two sites in Pakistan were 23 and 1/100,000 p-y respectively. By age group, peak age incidence was highly variable among the three countries, and across sites within the countries. For example, in Bangladesh, the highest incidence was reported among children aged 5–15 years, while in Nepal incidence peaked among 2–4 years old age group at one site, and 16–25 years old age group in the other. In Bangladesh, paratyphoid fever peaked among children <2 years of age. In all age-stratified estimates, high statistical uncertainty remained, and interpretation requires caution. The Surveillance for Enteric Fever in India (SEFI) study prospective community surveillance study component ran from 2017 to 2020 demonstrating that paratyphoid fever incidence in the 6 months through 14 years age group ranged from 8/100,000 *per* year in Vellore through 112/100,000 *per* year in Kolkata. Although underlying numbers were small, incidence peaked in the 10–14 years old age group in Vellore, Delhi, and Pune, and in the 5–9 years old age group in Kolkata. The SEFI hybrid surveillance study component collected data from 2017 through 2020. Here, paratyphoid incidence among those aged <15 years was 696 and 115/100,000 *per* year in Chandigarh and Anantapur, respectively, with no paratyphoid fever identified among hospitalized patients at the other sites in this age group. Incidence among those aged 15 years age or older was 437, 34, 25, 8, and 32/100,000 *per* year in Chandigarh, East Champaran, Nandurbar, Karimganj, and Kullu, respectively [61]. Finally the TSAP and the SETA surveillance studies conducted in Africa appear found that *S.* Paratyphi A disease was rare at the study sites [61]. In terms of age distribution, data from the IHME GBD estimates for 2021 indicate that paratyphoid fever is rare in the neonatal period, but it is estimated to peak at 12 months of age with an annual incidence of 116*/*100,000 p-y. The same model estimated an overall annual incidence of 27/100,000 p-y and showed a considerable progressive decline between 1990 and 2021. This decline is accompanied by greater statistical accuracy of estimates of incidence associated with increased availability of data [11]. The heterogenicity of findings across and within studies with regards to *S.* Paratyphi A disease highlights the need for accurate, good quality data on the incidence of disease and, particularly, age- stratified data.

With regards to antimicrobial resistance, while multidrug resistant *S.* Paratyphi A appears to have declined since the 1990s. By 2019, the prevalence of fluoroquinolone non-susceptibility (FQNS) ranged from 67 to 97 % in South Asia, Southeast Asia, east Asia, and Oceania and, despite remaining uncommon, an upward trend in resistance to third-generation cephalosporins was also observed [12].

## 3 Vaccines landscape and value proposition in context of endemic settings

Following the 2019 PDVAC recommendation [62], a vaccine value profile for *S.* Paratyphi A vaccines was published in 2023 [63]. The report emphasized the need for developing and deploying bivalent *S.* Typhi/Paratyphi A vaccines in south Asia, where the contribution of paratyphoid fever incidence in relation to overall enteric incidence appears to be increasing, and AMR rates are rising. It also identified several evidence and research gaps, including a lack of high-quality epidemiological data, insufficient burden of disease information, and the need for better diagnostic tools and treatment strategies in the face of evolving AMR patterns.

In the absence of blood culture and *Salmonella* serotyping or, alternatively, reliable, affordable, and easy-to-implement diagnostics, the term “enteric fever” may be wholly attributed to typhoid fever, and the contribution of paratyphoid disease overlooked. Moreover, even when blood culture is done successfully, *Salmonella* serotyping is seldom performed, hence paratyphoid fever cases due to serovars Paratyphi A, B, and C are not differentiated, and paratyphoid fever is commonly assumed to be caused by *S.* Paratyphi A. Hence, in the absence of accurate burden of disease data, advocating for, designing, and implementing effective targeted interventions against paratyphoid fever is often deprioritized in favour of a focus on typhoid fever alone. The distinction between typhoid and paratyphoid fever-causing serovars rely on isolation of the bacterium from blood, which is challenging in LMIC settings, has a low sensitivity of approximately 50 %, and is highly dependent on sampling technique, blood volume, and timely sample processing [[64], [65], [66], [67], [68]]. There is, therefore, a need for more effective culture-based methods and for the development of rapid and less complex testing methods, including molecular techniques and lateral flow point-of-care tests to address the diagnostic gap [69,70].

Several critical immunological knowledge gaps are impeding vaccine development. Foremost is the lack of defined correlates of protection – measurable immune markers or surrogates that can reliably predict vaccine efficacy in clinical trials. For instance, while current *S.* Paratyphi A- containing vaccines under development depend on the O-antigen of LPS being immunogenic and able to confer protection through the induction of serum IgG to O:2, this protective mechanism remains unproven. Furthermore, the limited understanding of broader immune response presents a challenge. The contribution of different antibody classes (*e.g.* IgA or IgM), the role of mucosal immunity, and the involvement of cell-mediated immunity require further investigation, particularly as the LPS O-antigen is a T -independent antigen, suggesting that other immune pathways or antigens may be crucial for effective, long-lasting protection. These gaps underscore the need for comprehensive immunological studies to inform the design and evaluation of effective vaccines.

A second gap is the lack of alignment on an acceptable regulatory pathway for licensure and registration. This includes determining the role of the CHIM in the absence of a classic phase 3 field efficacy trial, and whether licensure of *S.* Paratyphi A-containing vaccines could be achieved solely on the basis of noninferiority to currently licensed TCVs and considering the *S.* Paratyphi A component as adding valence to the vaccine in similar way that pneumococcal conjugate vaccines do. Countries in Asia, where paratyphoid fever poses a substantial burden, recognize the need for a vaccine to prevent a disease with similar severity to typhoid fever. Phase 1 clinical studies for bivalent *S.* Typhi/Paratyphi A live-attenuated oral vaccines and conjugate vaccines are ongoing or recently completed, and phase 2 safety and immunogenicity studies in India, paired with CHIM studies among adults in the UK, are to start in 2025 for at least one conjugate product [71]. For the live-attenuated vaccine candidate, a CHIM in UK adults is nearing completion [72,73]. National regulatory authorities (NRA) in endemic countries are open to grant licensure based on CHIM findings once data on the safety and reactogenicity of the products in an endemic target population are available, even if these studies are conducted in other countries.

Four WHO-prequalified *S.* Typhi conjugate vaccines use the Vi antigen conjugated to different protein carriers: TypBarTCV (Vi-TT), TyphiBev (Vi-CRM_197_), and more recently SKYTyphoid (Vi-DT) and ZyVac-TCV (Vi-TT). These typhoid conjugate vaccines could be leveraged to add an additional *Salmonella Paratyphi* A LPS O:2-antigen component conjugated to a carrier, either the same or different to the one already contained in the existing TCV component. However, a key challenge is achieving long-lasting protective immunity against *S.* Paratyphi A with a single dose and without the use of an adjuvant, as none of the currently WHO-prequalified TCV products include adjuvants. There is also potential to add other invasive non-typhoidal *Salmonella enterica* serovars (NTS), namely *S.* Typhimurium and *S.* Enteritidis, especially as the majority of *Salmonella* disease in the under-five year olds in Africa is caused by these two nontyphoidal serovars, and there are already some ongoing efforts in this direction [63,74,75]. Region-specific products, *i.e.,* bivalent enteric fever vaccines for Asia and NTS vaccines targeting *S.* Typhimurium and *S.* Enteritidis for Africa, are in development and expected available before a quadrivalent vaccine. These products are likely to be favoured in the market, as adding additional antigens would significantly increase costs, making more comprehensive formulations less economically viable [49].

The current *Salmonella* vaccine development pipeline is progressing steadily. For *S.* Paratyphi A, a live-attenuated vaccine, the CVD1902 produced by the University of Maryland in collaboration with Bharat Biotech has been tested in Oxford using a CHIM, and the results are currently awaiting publication. The current focus is, however, on bivalent *S.* Typhi/Paratyphi A conjugate vaccines. Two vaccine candidates are currently ready to enter phase 2/3 trials: Typhibev+O:2-CRM, developed by Biological E and GVGH, and a Vi-TT + O:2-DT from the Serum Institute of India, which has recently published the phase 1 study results [50]. There is a general consensus in the public health community that *S.* Paratyphi A vaccination will likely be delivered as a bivalent combination with *S.* Typhi, especially given concerns about the growing number of infant vaccinations in the infant immunization schedule.

## 4 Use of controlled human infection model for the evaluation of *S.* Paratyphi A vaccines and ascertainment of protection

Given the limited geography and fewer cases of paratyphoid A fever relative to typhoid fever, the feasibility of conducting phase 3 field efficacy studies likely will be challenging. In the absence of correlates of protection against *S.* Paratyphi A infection, alternatives are needed to ensure suitable efficacy data are made available to regulatory authorities to be able to issue a recommendation for vaccine licensure. CHIMs can be used to evaluate vaccine efficacy, and provide valuable insights into the development of the immune response, including evaluation of correlates of disease protection [76]. A *S.* Typhi CHIM was paramount to provide the necessary evidence to support the WHO prequalification of the first TCV by demonstrating that Typbar-TCV was able to induce a protective immunological response similar to that of VI-PS vaccines, and provided insights to the development of immunity and the potential correlates of protection against typhoid fever [77,78].

A CHIM model for *S.* Paratyphi A was established in 2014 [53,72], and the first evaluation of a live-attenuated vaccine against *S.* Paratyphi A is ongoing. Several existing knowledge gaps, such as host-pathogen interaction and the development of disease, immune protection, and duration of immune response, the identification of appropriate biomarkers of infection, correlates of protection, and vaccine efficacy against homologous and heterologous strain challenge remain unanswered [54]. The findings from the *S.* Paratyphi A CHIM so far appear to indicate that there might be some differences in disease severity and presentation between *S.* Typhi and *S.* Paratyphi A infection. Volunteers appear to be less symptomatic when infected with *S.* Paratyphi A than with *S.* Typhi despite the blood bacterial count being similar for both infections [53]. *Re*-challenging with *S.* Paratyphi A up to a year post-infection resulted in lower rates of infection than in the initial challenge, suggesting that prior exposure to the bacterium results in the development of some degree of protective immunity. Therefore, it is expected that a vaccine developed against *S.* Paratyphi A could be protective against infection. The *S.* Paratyphi A CHIM also corroborated that cross-protection between the two serovars is unlikely [54].

Currently, three vaccine candidates are most advanced in the development pipeline: an oral live-attenuated vaccine CVD1902; and two bivalent conjugate vaccines. All three are expected to evaluate efficacy in alignment with PDVAC recommendations [55]. For the CVD1902 product, CHIM findings are expected to be available in early 2025, and CHIM studies to evaluate the SII products are currently being set up.

When Phase 3 clinical efficacy trials are infeasible or attack rates are very low, leading to unpredictable timelines, CHIM studies offer a more efficient and cost-effective alternative to traditional phase 3 efficacy trials. However, the ethical challenge of deliberately exposing healthy volunteers to the disease must be carefully weighed together with the strength of evidence with respect to challenge strain and dose, and external validity of the results. In addition to assessing vaccine efficacy, CHIM studies allow for the collection of valuable samples to identify correlates of protection. For bivalent vaccines, the *S.* Typhi component can be licensed based on noninferiority to existing vaccines, which is straightforward in regions where TCVs are already approved. However, in areas where only polysaccharide typhoid vaccines are licensed, the acceptability of the immunobridging strategy needs further discussion. In any case, additional safety evaluations of the TCV component will be necessary. For the *S.* Paratyphi A component, CHIM studies can provide evidence of efficacy in controlled conditions. Collaboration between developers and regulators will be needed to ensure that postmarketing field effectiveness studies are conducted especially in countries where both diseases are present [76].

## 5 Regulatory considerations and pathway to licensure

A key challenge in the proposed regulatory pathway of a comprehensive vaccine against enteric fever, particularly in countries such as India and Bangladesh, is the fact that CHIM studies are not part of their rules and regulations, though data from CHIM studies conducted abroad can be considered, as was the case with TCV [79]. The standard regulatory approach would involve a phase 1 study to establish safety and reactogenicity, and an immunogenicity study in adult subjects, followed by phase 2 and phase 3 studies, in age descending cohorts including concomitant administration studies with other vaccines as *per* immunization schedule. A phase 2 trial would use a randomized design comprising age de-escalation, and a phase 3 field efficacy would use a randomized controlled trial design with an appropriate comparator vaccine. There would also be an expectation that a data safety and monitoring board (DSMB) is established for ongoing monitoring of adverse events. Last but not least, there is a need for analytically validated immunological assays to allow for the evaluation of vaccine-induced functional immune responses against the targeted pathogens. CHIM studies are not currently authorised to be conducted in some key *S.* Paratyphi A endemic countries, and such studies cannot be included as pivotal clinical trials for the licensure of a new paratyphoid A-containing vaccine by the regulatory authority. Should a correlate of protection be identified, the Phase 3 clinical trial that includes and immunologically naïve population (*e.g.* infants <2 years) could be designed with an immunogenicity endpoint, and regulators may include the commitment for a postmarketing effectiveness study at the time of marketing authorisation as a requirement to grant licensure. For licensure in India, safety would also have to be established for any new bivalent typhoid/paratyphoid vaccine in the endemic setting in phase 3 clinical safety trials, including in the target age groups across childhood. Data from CHIM studies, even if obtained in a non-endemic country such as the UK, will be key to support licensure in endemic settings such as India, and to facilitate the WHO prequalification processes.

The use of a bivalent *S.* Typhi/Paratyphi A vaccine in non-endemic settings would be mostly targeted to the travellers' market. In countries such as the UK, where TCVs are not licensed, the regulatory approval of a conjugated, bivalent *S.*Typhi/Paratyphi A product would require considerations for both components of the vaccine. Similarly to the approval in countries where these pathogens are endemic, the market authorisation of such a vaccine would require demonstration of noninferiority to the available typhoid licensed vaccines for the typhoid component, evidence of immunogenicity of the paratyphoid component, and evidence of protection against *S.* Paratyphi A infection, which could come from a CHIM study. Additionally, licensure in a high-income setting typically would require demonstration of vaccine safety for 6 months after last dose in at least 3000 participants, and immunogenicity to be shown through validated assays for both Vi and O:2 serum IgG antibodies [80].

Regardless of income level of the setting, once the first vaccine is licensed, subsequent products could be licensed on the basis of immunobridging, provided the antigen and vaccine technology in the new product are similar to the first-licensed product. In any case, post-licensure vaccine effectiveness data will need to be generated to confirm the benefits in the real world in different settings and context of use.

## 6 Conclusions

The consultation concluded that the regulator pathway recommended by PDVAC - requiring immunological noninferiority of the typhoid component and demonstration of protective efficacy for the paratyphoid component through CHIM studies, alongside field immunogenicity and safety trials in endemic settings - is likely to be accepted by NRAs in both: endemic countries and non-endemic countries. This pathway would accommodate vaccines intended for specific at-risk populations, and the travellers' market in non-endemic settings. However, it is important to note that in settings where TCVs are not licensed, data on the *S.* Typhi component might have to be compared with the unconjugated polysaccharide typhoid vaccines, depending on preferences from NRAs. For licensure in all settings, the bivalent product should have a favourable safety and immunogenicity profile. Immunobridging might be acceptable for the TCV component of the vaccine. However, data from a CHIM will be important to help contribute to confidence in *the S.* Paratyphi A component. A Phase I trial in a non-endemic site to ascertain safety and immunogenicity could facilitate evaluation in endemic settings. The CHIM might also provide data on the level of antibody responses associated with protection, and immune markers for that protection. In such circumstances, postmarketing studies are to be expected as requirements by national regulatory authorities in endemic and non-endemic countries. Licensure and regulatory approval requirements may differ by country and are guided by national regulations, which need to be considered during vaccine development to ensure all data are collected throughout the product development cycle and made available when required. Consultation with regulators should be planned from early development, and frameworks for fostering international regulatory convergence should be established. The commitment from manufacturers to postmarketing studies to ascertain vaccine effectiveness is important, as the CHIM cannot provide certainty at this time about the level of protection in target populations in LMICs.

In conclusion, the ongoing discussions on the development and licensure of a *S.* Typhi/Paratyphi A vaccine reflect a collective effort from academic groups, manufacturers, vaccine developers, and regulatory bodies to navigate the complexities of developing, licensing, and recommending a vaccine for which the classic regulatory pathway with a large efficacy phase 3 trial would not be feasible and CoPs are not yet established. This conversation emphasizes the need for innovative regulatory approaches, international collaboration, and comprehensive postmarketing studies. As regulatory approval requirements may differ by country and are guided by national regulations, it is advisable that consultation with regulators are planned from early development, and frameworks for fostering international regulatory convergence should be established.

## CRediT authorship contribution statement

**Ana Belen Ibarz-Pavon:** Writing – original draft, Validation, Project administration, Conceptualization. **Marie-Christine Bielsky:** Writing – review & editing, Validation. **Rubina Bose:** Writing – review & editing, Validation. **Marco Cavaleri:** Writing – review & editing, Validation. **John A. Crump:** Writing – review & editing, Validation. **Joachim Hombach:** Writing – review & editing, Validation. **David C. Kaslow:** Writing – review & editing, Validation. **Farhana Khaman:** Writing – review & editing, Validation. **Calman A. MacLennan:** Writing – review & editing, Validation. **Kirsty Mehring-Le Doare:** Writing – review & editing. **Andrew J. Pollard:** Writing – review & editing, Validation. **Firdausi Quadri:** Writing – review & editing, Validation. **Jacob John:** Writing – review & editing, Validation. **Annelies Wilder-Smith:** Writing – review & editing, Validation, Supervision, Conceptualization.

## Disclaimer

The authors alone are responsible for the views expressed in this paper and they do not necessarily represent the views, decisions, or policies of the institutions with which they are affiliated. The manuscript includes personal views of MC and must not be understood or quoted as being made on behalf of or representing the position of the European Medicines Agency or one of its committees or working parties.

## Declaration of competing interest

The authors declare that they have no known competing financial interests or personal relationships that could have appeared to influence the work reported in this paper.

## Data Availability

No data was used for the research described in the article.
